# Isolation and characterization of bacteriophages for controlling *Rhizobium radiobacter* – causing stem and crown gall of highbush blueberry

**DOI:** 10.3389/fmicb.2024.1437536

**Published:** 2024-08-02

**Authors:** Bowornnan Chantapakul, Siva Sabaratnam, Siyun Wang

**Affiliations:** ^1^Food, Nutrition and Health, University of British Columbia, Vancouver, BC, Canada; ^2^Abbotsford Agriculture Centre, Ministry of Agriculture and Food, Abbotsford, BC, Canada

**Keywords:** bacteriophage, gall disease, *Rhizobium radiobacter*, blueberry, biocontrol, genomic analysis

## Abstract

**Introduction:**

Stem and crown gall disease caused by the plant pathogen *Rhizobium radiobacter* has a significant impact on highbush blueberry (*Vaccinium corymbosum*) production. Current methods for controlling the bacterium are limited. Lytic phages, which can specifically target host bacteria, have been widely gained interest in agriculture.

**Methods:**

In this study, 76 bacteriophages were recovered from sewage influent and screened for their inhibitory effect against *Rhizobium* spp. The phages were genetically characterized through whole-genome sequencing, and their lytic cycle was confirmed.

**Results:**

Five potential candidate phages (isolates IC12, IG49, AN01, LG08, and LG11) with the ability to lyse a broad range of hosts were chosen and assessed for their morphology, environmental stability, latent period, and burst size. The morphology of these selected phages revealed a long contractile tail under transmission electron microscopy. Single-step growth curves displayed that these phages had a latent period of 80–110 min and a burst size ranging from 8 to 33 phages per infected cell. None of these phages contained any antimicrobial resistance or virulence genes in their genomes. Subsequently, a combination of two-, three- and four-phage cocktails were formulated and tested for their efficacy in a broth system. A three-phage cocktail composed of the isolates IC12, IG49 and LG08 showed promising results in controlling a large number of *R. radiobacter* strains *in vitro*. In a soil/peat-based model, the three-phage cocktail was tested against *R. radiobacter* PL17, resulting in a significant reduction (*p* < 0.05) of 2.9 and 1.3 log_10_ CFU/g after 24 and 48 h of incubation, respectively.

**Discussion:**

These findings suggest that the three-phage cocktail (IC12, IG49 and LG08) has the potential to serve as a proactive antimicrobial solution for controlling *R. radiobacter* on blueberry.

## 1 Introduction

*Rhizobium radiobacter*, formerly known as *Agrobacterium tumefaciens/A. radiobacter*, has been identified as the causative agent of stem and crown gall disease in blueberries (Kuzmanović et al., 2019). The general characteristics of *R. radiobacter* are gram-negative rod-shaped bacterium and generally found in soil environments and roots of plants (Guo et al., 2017). Most plant-pathogenic *Rhizobium* species possess a tumor-inducing plasmid (pTi) responsible for developing gall in wounded plant cells. The transfer DNA (T-DNA) segment of pTi plasmid, upon integration into plant genome, results in the imbalance of plant growth hormones such as auxin and cytokinin (Gohlke and Deeken, 2014). Once the equilibrium of plant hormones is disrupted, the abnormal growth of cells develops into galls that resemble tumors (Kunkel and Harper, 2018). Gall, typically seen as ball-shaped or knob-like structures on various parts of plants such as roots, crowns and stems or even flowers and stems, can obstruct the vascular system of plants, resulting in obstruction of conducting water and nutrients beyond the gall’s location (Aloni and Ullrich, 2008). While galls typically do not cause serious effects on the adult plant, they can have significant impacts on young blueberry plants (Pulawska, 2010). Although cases related to stem gall disease in blueberries were rarely found until recently, blueberry farmers in British Columbia have reported incidents and requested a solution to minimize the impact of gall disease. In addition, the Canadian blueberry industry, being one of the World’s largest suppliers, faces economical loss from stem and crown gall disease, estimated at $11.2 million per year (Carter, 1989).

The method for controlling gall disease involves the use of chemicals such as copper and acids (Sawalha et al., 2013; Mary Opisa et al., 2020). However, heavy use of these chemicals can contribute to soil pollution, the development of resistant strains, and potential harm to humans. Commercial biocontrol therapies have been available for decades to prevent *R. radiobacter* in some plants. For instance, *A. radiobacter* strain K84 has been shown to produce bacteriocin called agrocin 84 and used to control *A. tumefaciens* in cherry (Stockwell et al., 1993; Penyalver et al., 2001). While this inhibitory compound is very effective against *R. radiobacter*, the application may adversely affect beneficial microbes that are closely related to other *Rhizobium* species. To date, there is no effective method for curing stem gall disease in highbush blueberry. The best strategy for controlling gall disease is proactive prevention before the onset of the disease.

The use of bacteriophage (phage) has perceived the interest in plant disease management due to their unique trait that overcomes other biocontrol methods. Phage specifically binds to bacterial host cells through a particular receptor, leading to the lysis of specific target bacterial hosts. In other words, phage cannot attach to nonbacterial hosts and rarely any evidence of cross-binding to other genera, minimizing the risk of damage to non-target and beneficial species. Over the past decades, successful developments of phage treatments in agriculture have demonstrated their ability to outperform other biocontrol strategies. Phages have been used to control soft rot in potato (Adriaenssens et al., 2012; Czajkowski et al., 2015), bacterial wilt in tomato (Fujiwara et al., 2011), bacterial spot in citrus (Balogh et al., 2008), and bacterial blight in leek (Rombouts et al., 2016). In the context of phage biocontrol targeting gall disease-causing bacteria, phage Atu_ph02, isolated from wastewater, showed the ability to subdue *Agrobacterium* in potato discs (Attai et al., 2017). Recently, phage OLIVR 1 to 6, isolated from tomato greenhouses, demonstrated the inhibitory effect against *Agrobacterium* biovar 1 strain in a hydroponic solution (Fortuna et al., 2023). In addition, a commercially successful phage treatment, AgriPhage™, has been used in agricultural field to control *Xanthomonas campestris* or *Pseudomonas syringae* in Tomato (Ly-Chatain, 2014). However, in the case of gall disease, neither phages nor chemicals can penetrate the shell of galls once they have developed. Phages, however, manifest potential as biocontrol agents because they can enter through the roots of the plants and be applied directly to soil to inhibit target pathogens (Buttimer et al., 2017). In addition, to the best of our knowledge, the application of phage biocontrol to manage stem gall disease-causing bacterium in blueberry has not been explored. Therefore, the objectives of this study were to optimize isolation techniques and isolate phages from natural environment, characterize the selected phages for efficacy against *R. radiobacter*, and evaluate their inhibitory effect against *R. radiobacter* in a soil/peat-based system.

## 2 Materials and methods

### 2.1 Bacterial strains and growth conditions

The *Rhizobium* strains, previously isolated from blueberry farms, were used as the host strains for phage isolation in this study (Table 1). In addition, *R. radiobacter* ATCC 25235 and *R. radiobacter* ATCC 33970 from the American Type Culture Collection (ATCC) were used as a control. All strains were stored at −80°C in nutrient broth (NB; BD/Difco, East Rutherford, NJ, United States) supplemented with a final concentration of 20% glycerol. For working stocks, all *R. radiobacter* strains were grown at 28 ± 2°C in nutrient agar (NA; BD/Difco, East Rutherford, NJ, United States). For each experiment, new sets of bacterial cultures were prepared by inoculating a single colony into 10 mL of NB and incubating at 28 ± 2°C and 150 rpm for 24 h on an orbital shaker (311DS, Labnet, Edison, NJ, United States).

**Table 1:** *Rhizobium* strains used in this study.

Strain	Species	Source
Bn18	*Rhizobium rhizogenes*	Blueberry
Bn37	*Rhizobium radiobacter*	Cucumber
Bn38	*Rhizobium radiobacter*	Cucumber
Bn42	*Rhizobium rhizogenes*	Blueberry
Bn46	*Rhizobium rhizogenes*	Blueberry
14736-1	*Rhizobium rhizogenes*	Blueberry
14736-2	*Rhizobium rhizogenes*	Blueberry
M11	*Rhizobium rhizogenes*	Blueberry
M13	*Rhizobium rhizogenes*	Blueberry
Edr1-6	*Rhizobium rhizogenes*	Blueberry
M22	*Rhizobium rhizogenes*	Blueberry
M26	*Rhizobium rhizogenes*	Blueberry
M29	*Rhizobium rhizogenes*	Blueberry
M30	*Rhizobium rhizogenes*	Blueberry
M33	*Rhizobium radiobacter*	Blueberry
M35	*Rhizobium rhizogenes*	Blueberry
PL3	*Rhizobium radiobacter*	Blueberry
PL8	*Rhizobium rhizogenes*	Blueberry
PL9	*Rhizobium rhizogenes*	Blueberry
PL10	*Rhizobium rhizogenes*	Blueberry
PL11	*Rhizobium rhizogenes*	Blueberry
PL12	*Rhizobium rhizogenes*	Blueberry
PL13	*Rhizobium rhizogenes*	Blueberry
PL14	*Rhizobium rhizogenes*	Blueberry
PL15	*Rhizobium rhizogenes*	Blueberry
PL16	*Rhizobium rhizogenes*	Blueberry
PL17	*Rhizobium radiobacter*	Blueberry
25235S	*Rhizobium radiobacter*	Potato
25235L	*Rhizobium radiobacter*	Potato
33970	*Rhizobium radiobacter*	Cherry gall

### 2.2 Phage isolation and purification

Isolation of phages was performed with modifications of previously published protocols (Mendum et al., 2001; Halmillawewa et al., 2015; Fong et al., 2017). Strains of *R. radiobacter*, as listed in Table 1, including *R. radiobacter* ATCC 25235 and *R. radiobacter* ATCC 33970, were used as hosts for the isolation of phages. Briefly, 20 g of samples from the soil collected from the vicinity of stem gall infected blueberry plants, 20 mL water samples from drainage ditches in stem gall infected blueberry farms, and 20 mL of sewage influent from waste water treatment plant in metro Vancouver region were prepared by adding 20 mL of 2X NB and a cocktail of 400 μL of sets of five overnight cultures of *Rhizobium* strains of the 28 strains at a time as hosts were placed in 50 mL centrifuge tubes. The samples were incubated in a shaker at 150 rpm and 28 ± 2°C for 24 h. Then, the supernatant was centrifuged at 4,000 *x g* for 10 min and filtrated through a 0.45 μm filter (Cytiva, Marlborough, MA, United States). A 100 μL aliquot of the filtrate was mixed with 300 μL of an overnight culture of each of the 28 *Rhizobium* strains and 4 mL of 0.7% NB agar in 90 mm Petri dish using the double agar overlay technique (Kropinski et al., 2009).

The plates were incubated at 28 ± 2°C for 24–48 h to visualize plaque activity in the bacterial colonies. Upon observing the presence of plaque activity, plaques were removed from the agar surface using a sterile inoculating loop and suspended in 200 μL of SM buffer (100 mM NaCl, 8 mM MgSO_4_.7H_2_O, 50 mM Tris-Cl pH 7.5, and dH_2_O). The suspension was left at room temperature for at least 6 h. Double agar overlay technique was used with the suspension from the previous step for phage purification. To obtain a pure plaque, this step was repeated at least 5 times, and the purified phages were stored in SM buffer at 4°C for long-term storage.

### 2.3 Host range assessment

The assessment of host range of the isolated phages (76 isolates) was performed as described by Kutter (2009) with some modifications. Phage ATCC 25235b and 25236b were served as positive controls as they have been proven to target *R. radiobacter*. Briefly, 5 μL aliquots of ∼10^6^ PFU/mL of each of the 76 phages from the phage collection were spotted on the lawn of *Rhizobium* host in duplicate in Petri dish. The phage droplets were allowed to air dry under a biosafety cabinet and then incubated at 28 ± 2°C. After 24 h, the zones of lytic activity on the lawn of *Rhizobium* hosts were recorded using a scale of 0–4, where 0 = no activity and 4 = complete lysis. The assessment of host range and lytic activity of the phages was done in triplicate.

### 2.4 Lysis efficacy of *Rhizobium* phages in broth

The overnight-grown cultures of *R. radiobacter* hosts were prepared as previously mentioned in the section 2.1 “Bacterial strains and growth conditions.” After centrifugation at 4000 ×*g* for 10 min, the cell pellets were washed three times with fresh NB and adjusted to a final concentration of 5 × 10^6^ CFU/mL. Then, 50 μL aliquots of the cultures were transferred into a 96-well plate and treated with the 12 selected phages (IG09, IG14, IC12, IG49, IG57, IG58, AN01, LL05, LL09, LG08, LL11, and LL10) at the multiplicity of infection (MOI) of 100 PFU/CFU. Plates were placed into a plate reader (SpectraMax M2, Molecular Devices, Sunnyvale, CA, United States) set to 25°C and the cell density measurements (OD_600_) were taken every 6 h for 48 h. Each experiment was independently done in duplicate.

### 2.5 Transmission electron microscopy

Morphology of the phage isolates was identified using negative staining transmission electron microscopy (TEM). The method was performed as previously described by Alexeeva et al. (2018) with some modifications. Briefly, a high concentration of the phage lysates (~10^11^ PFU/mL) was centrifuged at 4°C for 1 h at 21130 *×g*. The supernatant was carefully discarded, retaining the last 100 μL of the phage suspension. Subsequently, 1.5 mL of 0.1 M sterile ammonium acetate was added to the suspension and centrifuged at 4°C for 1 h at 21130 *×g*. This step was repeated twice. After concentration, the purified phage suspension was applied to carbon coated copper grids (Ted Pella, Redding, CA, United States) followed by glow-discharge and then stained with 0.5% uranyl acetate. The phage images were taken using a Tecnai Spirit TEM (Fei company, Hillsboro, OR, United States) at an accelerating voltage of 80 kV.

### 2.6 Single-step growth curves

The single-step growth curve was used to determine the latent period and burst size of the phages in their respective hosts. Briefly, 1 mL of bacterial host corresponding to the phage amplification was diluted to 10^8^ CFU/mL using a calibration curve. Subsequently, the phages (IC12, IG49, AN01, LG08 or LG11) were individually added to an MOI equal to 0.01 or approximately 10^6^ PFU/mL. After allowing the phage to attach to the host for 5 min, unattached phages were removed by centrifuging at 4°C for 10 min at 4000 ×*g*. The supernatant was discarded, and a 1 mL aliquot of NB was added to resuspend the pellet. Immediately, the co-culture was incubated at room temperature and enumerated at 10-min intervals for 150 min. The assay was performed in triplicate.

### 2.7 Temperature and pH stability assay

The stability of the five most potential phages was examined in order to determine their viability under different pH and temperature conditions. For the pH stability test, the phages, IC12, IG49, AN01, LG08 and LG11, were diluted to ~10^6^ PFU/mL in SM buffers at pH 4, 6 and 8, and incubated at 22°C. Similar to the evaluation for the stability of the phages at various pH, phages were diluted in SM buffer at ~10^6^ PFU/mL and placed at 4, 22, and 37°C. To test the stability of the phage suspensions at −20°C, phages were aliquoted into 100 μL portions and placed in microcentrifuge tubes to prevent the freeze–thaw cycle. The concentrations of the phages were assessed on days 0, 1, 3, 5, 7, 10, 15, 20, 25 and 30. All experiments were done in triplicate.

### 2.8 DNA extraction and sequencing

Total genomic DNA from 71 phage isolates was extracted using the Norgen Biotek Phage DNA isolation kit (Norgen Biotek, ON, Canada) following the manufacturer’s specifications with some modifications. Briefly, 1 mL of high concentration of phage lysates (~10^9^–10^11^ PFU/mL) was treated with the DNase 1 kit (Norgen Biotek) to degrade host nucleic acid, following manufacturer’s instructions, and lysis buffer was added to the phage lysates and incubated at 65°C for 30 min. Afterward, the phage lysates were washed and eluted as per manufacturer’s instructions.

The DNA library was constructed using Nextera XT DNA Library Preparation Kit (Illumina, Hayward, CA, United States) according to manufacturer’s instructions and high-throughput sequencing was performed using Illumina MiSeq platform (Illumina).

### 2.9 Genome analysis

Contigs obtained from Illumina MiSeq were quality-checked using FastQC (Andrews, 2010) and low-quality reads were trimmed with Trimmomatic (Bolger et al., 2014). High quality paired-end reads were assembled into single circular contigs via Unicycler (Wick et al., 2016). The quality and %GC content of the genome assemblies were assessed using QUAST (Mikheenko et al., 2018). Open reading frames (ORFs) were identified and annotated with Pharokka using the Phanotate gene predictor (Bouras et al., 2022). Antimicrobial resistance genes and virulence genes were screened with ABRicate using Resfinder and VFDB database (Seemann, 2016), respectively. The phage life cycle was determined with Bacphlip (Hockenberry and Wilke, 2021). The completed phage genome was searched against other phage nucleotides using BLASTn of National Center for Biotechnology Information (NCBI) database. For phylogenetic tree, complete phage nucleotide sequences were aligned using ClustalW algorithms (Larkin et al., 2007) and the phylogenetic tree was constructed by the Maximum-Likelihood method using IQ-tree with 1,000 bootstrap replicates (Nguyen et al., 2014). The phylogenetic tree was visualized using iTOL (Letunic and Bork, 2021).

### 2.10 Application of a phage cocktail in artificially contaminated soil

A soil/peat-based (hereafter referred to as soil-based) *R. radiobacter* system was used to evaluate the efficacy of a phage cocktail (IC12, IG49, and LG08) *in vitro*. Soil-based substrate was obtained from Sun Gro Horticulture® (MA, United States) that comprised 65–75% Canadian Sphagnum peat moss, perlite, dolomite lime, and a wetting agent. An overnight culture of *R. radiobacter* PL17 was centrifuged at 4000 *x g* for 10 min and washed three times with NB. Parallelly, 1 g of the autoclaved soil-based substrate was added to a 50 mL centrifuge tube, and 5 mL of the *R. radiobacter* was inoculated to the soil-based substrate at a final concentration of 10^6^ CFU/mL. For the treatment group, an equal amount of phage cocktail (IC12, IG49, and LG08) or phage IG49 was added after the bacterial host reached an MOI of 100. Soil-based substrate added with 10 mL of 0.1% peptone water (PW) was used as a control group to check the background. In addition, the phage cocktail was added to the soil-based substrate as a phage quality control. Tubes were incubated at 22°C and 200 rpm for three days. At each 24-h interval, a 1-mL suspension was diluted in 0.1% PW and plated onto NA. Plates were incubated at 22°C- for 48–72 h and *R. radiobacter* colonies were enumerated. The experiment was done in triplicate.

### 2.11 Statistical analysis

All statistical tests were conducted using GraphPad Prism 9.2 (GraphPad Software, Inc., San Diego, CA, United States). For the phage stability tests, Student’s *t*-test was used to compare the means of two study groups. To compare multiple biocontrol treatments at the same time point, a one-way and two-way analysis of variance (one-way/two-way ANOVA) was conducted. All statistical analysis was performed at the significance level (α) at 0.05.

## 3 Results

### 3.1 Phage isolation and host range determination

A total of 105 phages based on the lysis of *Rhizobium* as host were isolated from the sewage samples, with only 76 phages (72.38% recovery rate) were successfully recovered and confirmed to be stable after five times purification and stored at 4°C at least 30 days. Notably, no phages were isolated from either soils or drainage water samples from gall disease-infected blueberry fields. The majority of the phages were recovered with the *Rhizobium* host M11, M13, M22, M30, M35, Edr1-6, PL8, PL9, PL14, PL15, and PL17.

**Figure 1:**
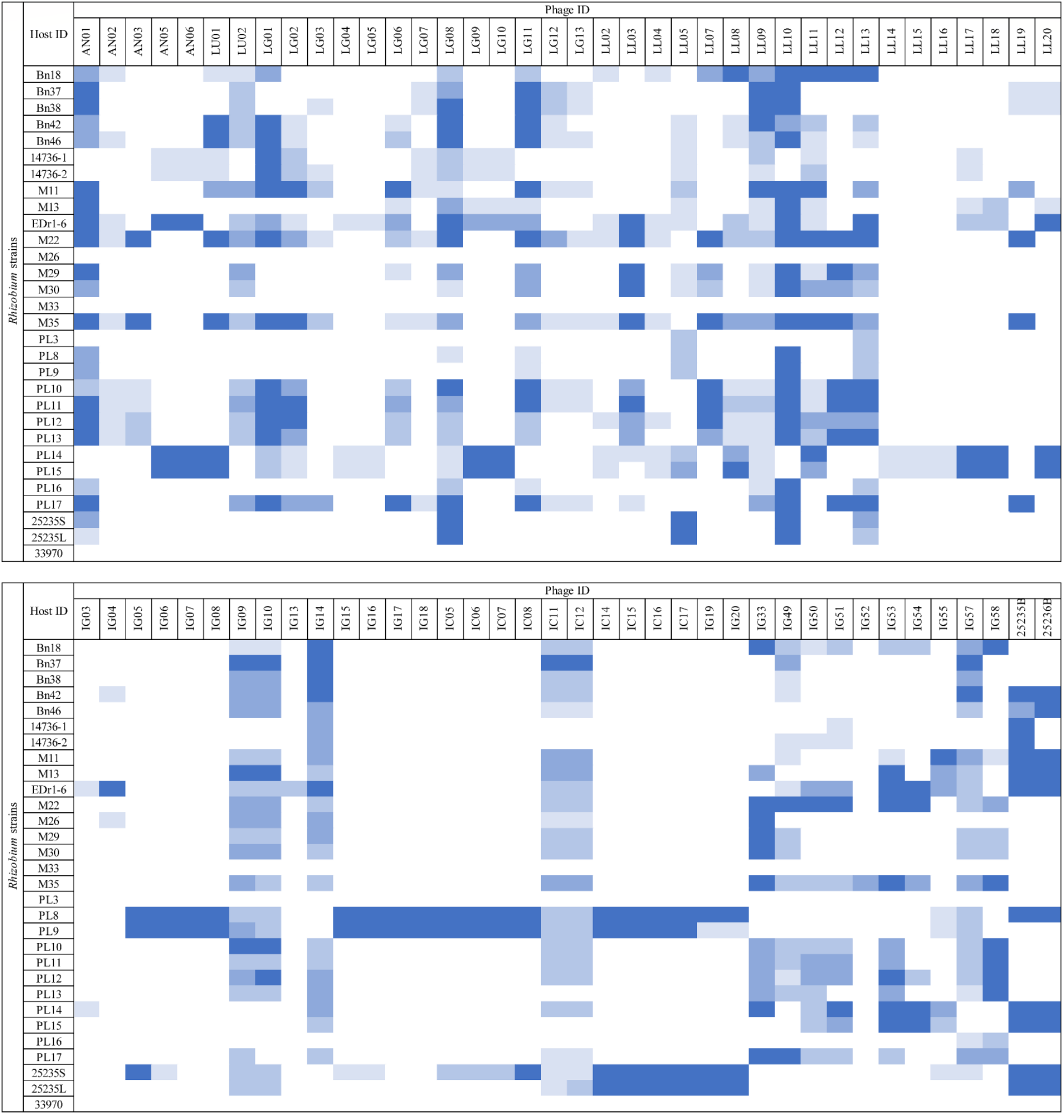
Host range assessment of phages. *Rhizobium* isolates susceptible to lytic phages were determined by the inhibition zones (□ = no lysis, 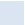 = low lysis, 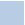 = medium lysis, 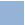 = almost complete lysis, and 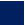 = complete lysis).

The host range of the 76 phage isolates was assessed against 28 strains of *Rhizobium*, including *R. radiobacter* ATCC 25235 and ATCC 33970 (Figure 1). Among these phages (*n* = 76), 18/76 (23.68%) showed the ability to lyse at least 15/28 (53.57%) *Rhizobium* strains and 10/76 (13.16%) phages exhibited the ability to lyse at least 21/28 (75%) *Rhizobium* strains. However, only two phages (LL05 and LL13) exhibited lytic ability against *R. radiobacter* isolate PL3. None of the phages were able to lyse *R. radiobacter* ATCC 33970 on the spot test. Based on the host range determination, only 12 phages were selected for further assessment.

### 3.2 Assessment of phages for lytic activity in broth

**Figure 2:**
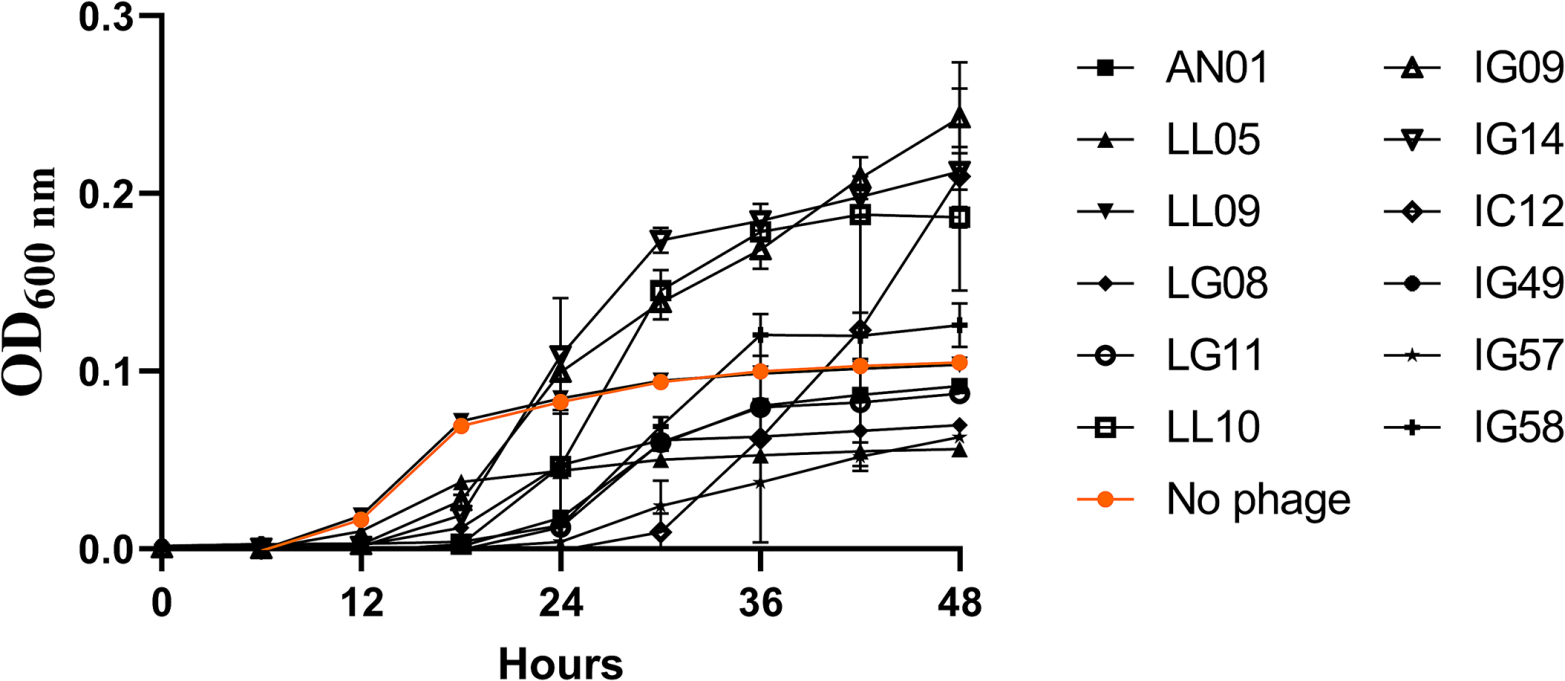
Single phage efficacy against *Rhizobium radiobacter* PL17 at MOI = 100.

In order to identify the most effective single phages for designing an effective phage cocktail against *R. radiobacter*, 12 individual phages were tested against *R. radiobacter* isolate PL17 at a MOI of 100 PFU/CFU at 25°C (Figure 2). The lytic activity of each phage was assessed based on its ability to either delay or inhibit the bacterial growth. The growth of *R. radiobacter* PL17 began to increase after 12 h. During that period, the treatment with phage LL09 did not suppress the growth of *R. radiobacter* PL17, while the other phages displayed inhibitory effects. By 18 h, treatments with single phage IG09, IG14, IC12, IG49, IG57, IG58, AN01, LG08, LG11 and LL10 showed a significant reduction (*p* < 0.05) when compared to the no-phage treatment. During the period of 24 h, the lytic ability of single phage IG09 and IG14 began to diminish, and no longer delayed the growth of *R. radiobacter* PL17. After 36 h, only the treatments with single phage IC12, IG49, IG57, LG08 and LL05 showed a significant decrease (*p* < 0.05) in the growth of *R. radiobacter* PL17. Only the treatment with phage IG49 completely inhibited the growth of *R. radiobacter* PL17 for 48 h, while the treatment with single phage IG57, LG08 and LL05 delayed the growth of the bacterium for 48 h. To qualitatively compare the inhibitory effect of each phage on the 28 *Rhizobium* isolates in a broth system, single phage-host efficacy graphs were converted to a heatmap, as shown in the Supplementary Figure S1.

**Figure 3:**
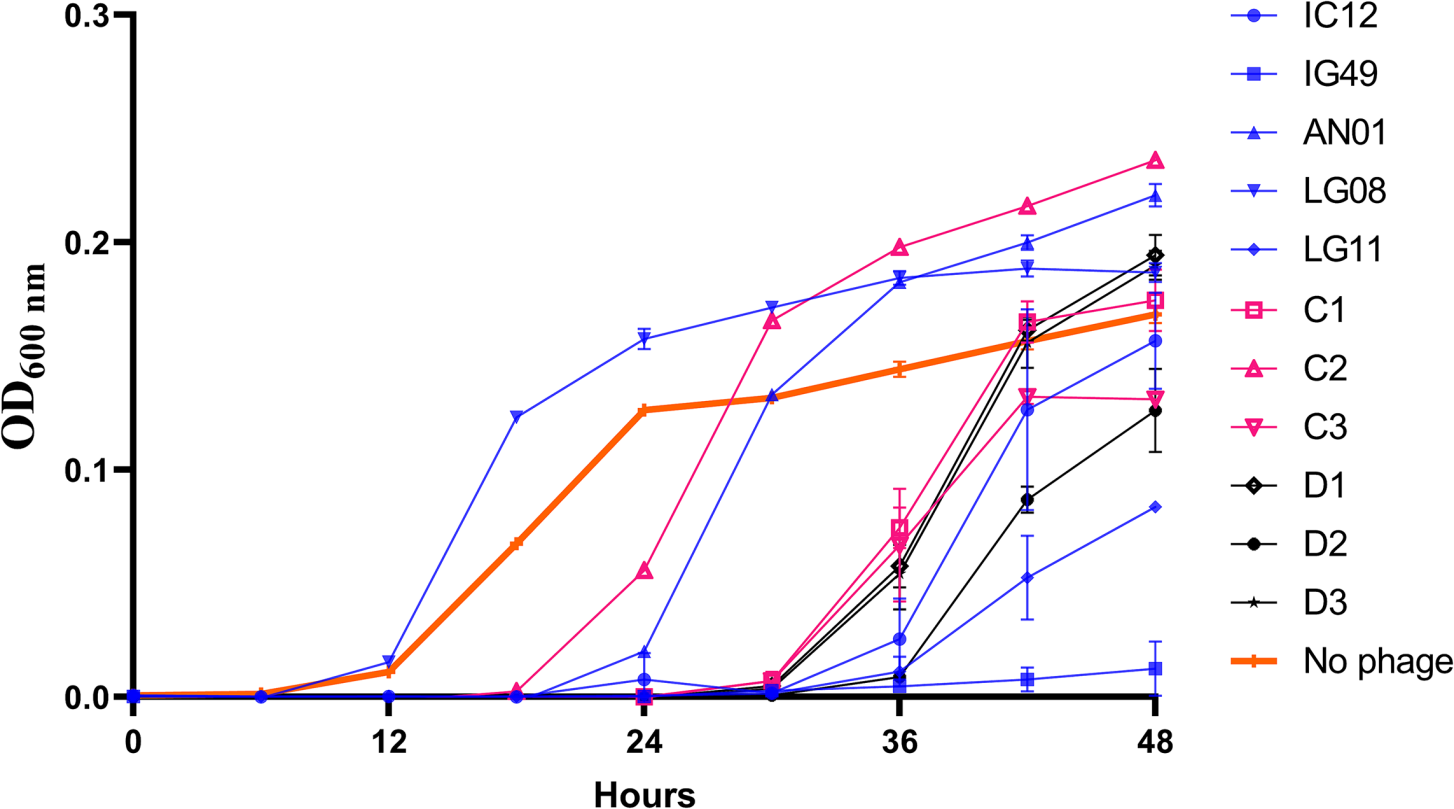
Efficacy of a combination of phage cocktail against *Rhizobium radiobacter* PL17 at MOI = 100. C1 – IG49, LG08 and IC12; C2 – IG49, LG08 and AN01; C3 – IG49, LG08 and LG11; D1 – IG49, LG08, IC12 and AN01; D2 – IG49, LG08, IC12 and LG11; D3 – IG49, LG08, AN01 and LG11. *Colored graph indicates the number of phages in each formulation. Blue is a single phage; Pink is a three-phage cocktail; Black is a four-phage cocktail.

To assess the synergistic effect of the phages in a broth-based system, various combinations of three- and four-phages cocktails consisting of the 5 most effective phages, based on the single phage efficacy results, were tested against *R. radiobacter* PL17 (Figure 3). All combinations of three- and four-phage cocktails were effective during the first 24 h, and thereafter the lytic activity began to decrease after 36 h. At 48 h; it appeared that only the treatments with three-phage cocktail (IG49, LG08, and LG11; C3) and the four-phage cocktail (IG49, LG08, IC12, and LG11; D2) were effective in delaying the growth of *R. radiobacter* PL17.

### 3.3 General characterization of phages

#### 3.3.1 Latent period and burst size

**Figure 4:**
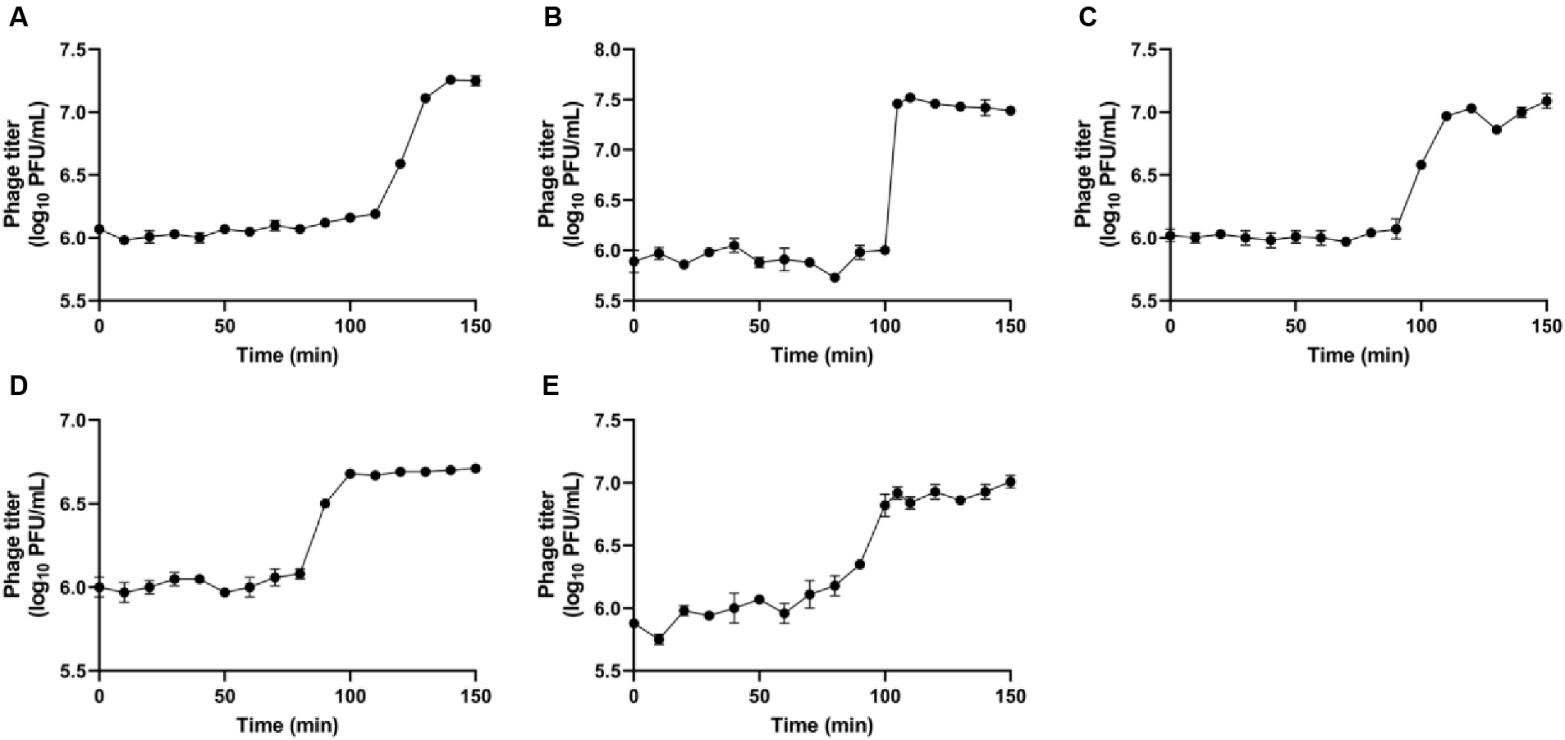
Single-step growth curves of the phages **(A)** IC12, **(B)** IG49, **(C)** AN01, **(D)** LG08, and **(E)** LG11. Data shown are the mean of three replicates ± SD.

The single-step growth curve was used to study the activity of phage amplification within the host cell, allowing to identify both latent period and burst size of the phages (Figure 4). These parameters are crucial for determining the most effective candidate phages to formulate different combinations of phage cocktail, aimed at assessing their efficacy against *R. radiobacter*. The latent period indicates the time taken for phages to propagate within the host cells, leading to the release of phase progenies upon cell lysis. The observed latent periods for the phages IC12, IG49, AN01, LG08 and LG11 were 110, 100, 90, 90 and 80 min, respectively. The burst size, calculated by the difference in the phage titer just after host lysis, determined the number of progenies produced per infected host cell. The burst size of IC12, IG49, AN01, LG08 and LG11 was determined as 14, 33, 9, 4 and 8 phages per infected host cell, respectively.

#### 3.3.2 pH and temperature stability

**Figure 5:**
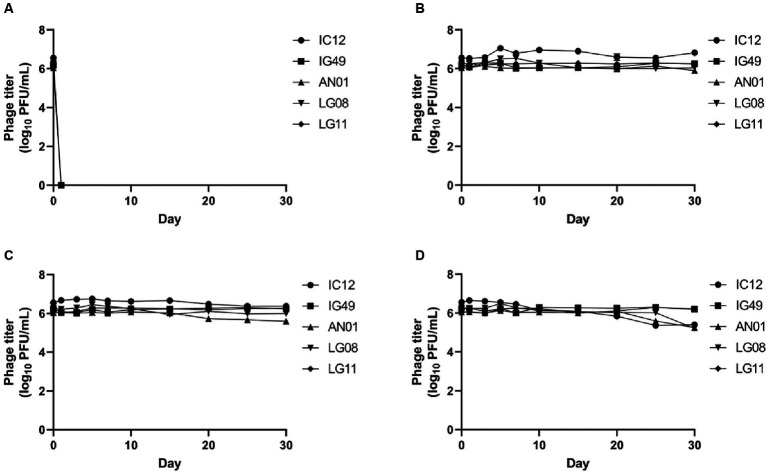
Stability of phage IG12, IC49, AN01, LG08, LG11 at **(A)** −20°C, **(B)** 4°C, **(C)** 22°C, and **(D)** 37°C over 30 days.

The survivability of phage IC12, IG49, AN01, LG08 and LG11 at different temperatures is shown in Figure 5. The 30-days storage period was chosen to assess the lytic activity over storage time and for potential phage application in field trials. None of the five phages were able to survive at −20°C (Figure 5A). Phages IC12, IG49, AN01, LG08 and LG11 were significantly (*p* > 0.05) stable at 4°C (Figure 5B) and 22°C (Figure 5C) for 30 days. However, at 37°C, the concentration of phage IC12, AN01 and LG08 was reduced by 1 log_10_ PFU/mL after 30-day of storage, while phages IG49 and LG11 remained significantly (*p* > 0.05) stable at 37°C (Figure 5D). These results suggested that these five phages remained stable at 4–37°C during 30 days of storage.

**Figure 6:**
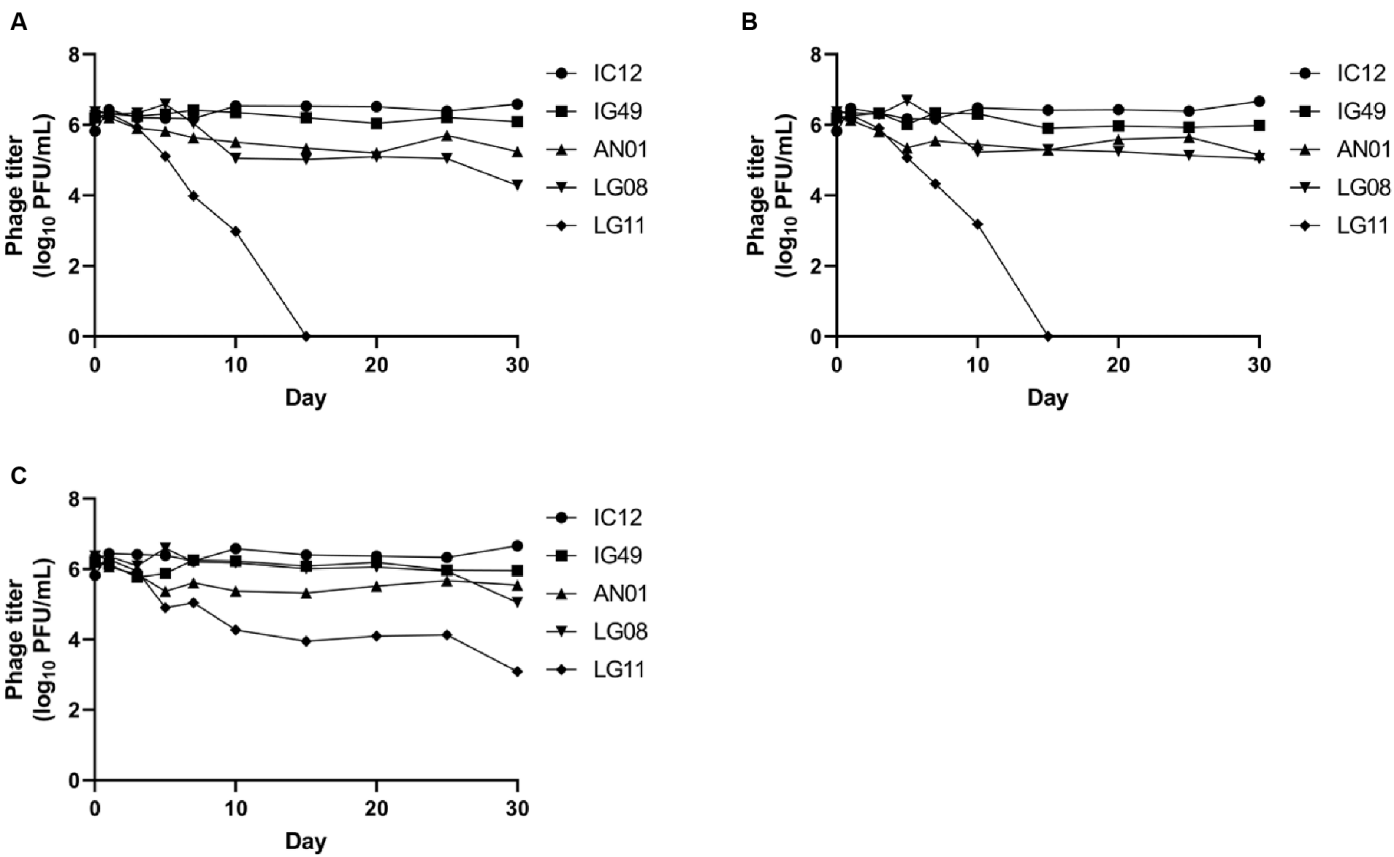
Stability of phage IG12, IC49, AN01, LG08, LG11 at **(A)** pH 4, **(B)** pH 6, and **(C)** pH 8 over 30 days.

The concentrations of phages IC12, IG49, AN01, LG08 and LG11 at pH 4, 6 and 8 after 30 days of storage are presented in Figure 6. At pH 4 and 6, phages IC12, IG49, and AN01 remained stable throughout the 30 days, while the concentration of phage LG08 slightly decreased by day 30. Phage LG11, however, declined from 10^6^ PFU/mL to 10^4^ PFU/mL starting on day 7 and thereafter rapidly declined to undetectable levels after 15 days. At pH 8, all phages except LG11 remained stable over 30 days. Phage LG11 significantly decreased (*p* < 0.05) from 10^6^ PFU/mL to 10^4^ PFU/mL after 10-day of storage, then remained stable until day 25, before decreasing to 10^3^ PFU/mL by the end of storage period. These results suggest that phage LG11 is the least stable biocontrol agent in acidic conditions, while other candidate phages remained stable in acidic conditions.

#### 3.3.3 Phage morphology under TEM

**Figure 7:**
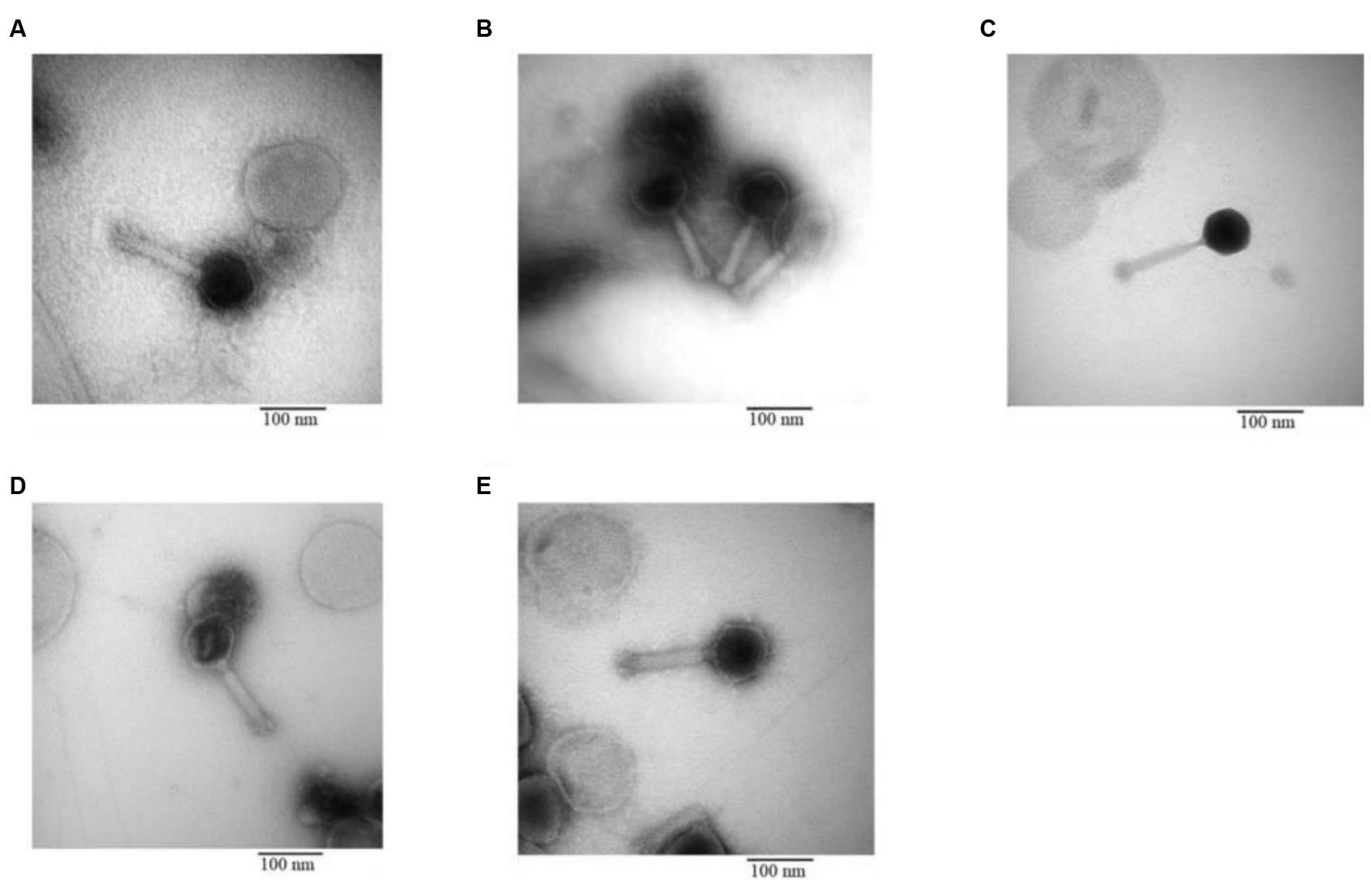
Transmission electron microscopy of phage **(A)** IC12, **(B)** IG49, **(C)** AN01, **(D)** LG08, **(E)** LG11.

The morphology of the phage was revealed through TEM. All five phages isolated from different sewage sources possessed the same structure: an icosahedral head and a long contractile tail, characteristics that classify them under the family *Myoviridae*. The specific length of the head and tail for each phage are illustrated in Figure 7.

### 3.4 General features of the phage genomes

The complete genome of 71 phages used in the formulation of three phage cocktails for potential biocontrol agents for *R. radiobacter* was obtained through whole-genome sequencing. Phage IC12 (Accession no. PP417939), IG49 (Accession no. PP429226), and LG08 (Accession no. PP429227) have circular dsDNA genomes with the sizes of 150,526 bp, 151,705 bp, and 149,984 bp, and the GC contents of 47.26, 46.86 and 47.12%, respectively.

Genome annotation using Pharokka revealed that phage IC12 contains 255 putative open reading frames (ORFs) and two tRNAs, with 60 annotated and 195 uncharacterized ORFs (Supplementary Figure S2A). These annotated ORFs were classified into four functional elements: phage structural and packaging-related proteins (22 ORFs), DNA-associated proteins (33 ORFs), lysis-associated protein (1 ORFs) and auxiliary genes (5 ORFs). Additionally, no integrase module was found in this phage genome. The genome sequence of phage IC12, aligned with NCBI database, revealed 93% coverage and 94.99% identity with *Agrobacterium* phage OLIVR5 (Accession no. NC_055841.1).

The genome of IG49 consists of 269 ORFs and two tRNAs, with 69 annotated and 200 uncharacterized ORFs (Supplementary Figure S2B). The predicted elements were categorized into four functional elements: phage structural and packaging-related proteins (24 ORFs), DNA-associated proteins (38 ORFs), lysis-associated protein (1 ORFs) and auxiliary genes (6 ORFs). Phage IG49 does not contain any lysogeny-related module such as integrase. The genome of phage IG49 was aligned to *Agrobacterium* phage OLIVR5 (Accession no. NC_055841.1) with the highest of 95% coverage and 96.64% identity.

The genome of LG08 contains 256 ORFs and two tRNAs, with 63 annotated and 193 uncharacterized ORFs (Supplementary Figure S2C). These annotated ORFs were divided into four functional elements: phage structural and packaging-related proteins (24 ORFs), DNA-associated proteins (32 ORFs), lysis-associated protein (1 ORFs) and auxiliary genes (6 ORFs). Phage LG08 also does not contain any lysogeny-related module. The genome of phage LG08 was aligned to *Agrobacterium* phage OLIVR5 (Accession no. NC_055841.1) with 92% coverage and 93.46% identity.

### 3.5 Phylogenetic analysis

**Figure 8:**
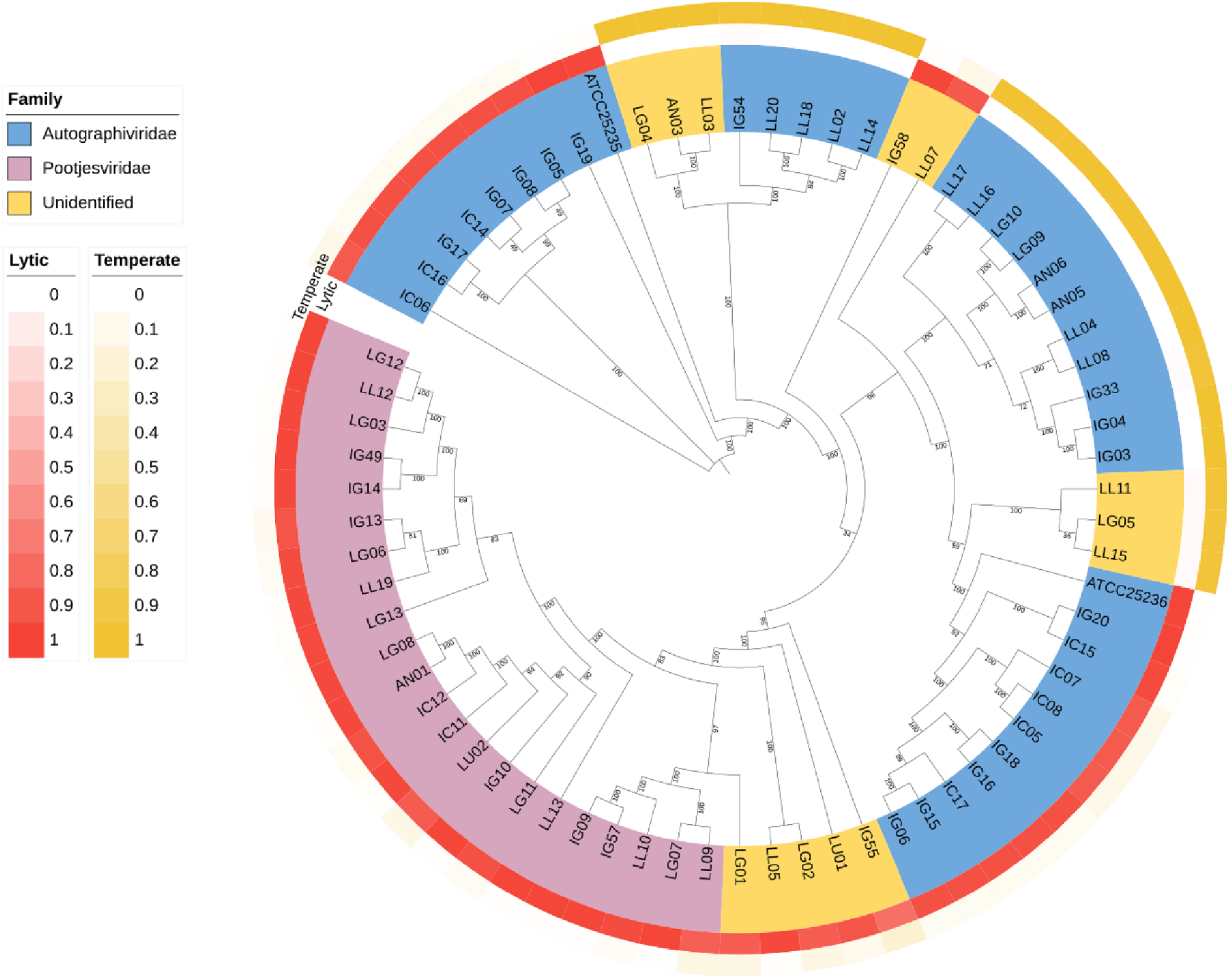
Phylogenetic tree of the whole genome sequencing of 71 isolates of phages. The genomic sequences were aligned using ClustalW alignment and the tree was constructed using Maximum-Likelihood method using IQ-TREE with 1,000 bootstrap replicates.

A Phylogenetic tree was constructed using whole-genome sequencing data from 71 isolates of phages (Figure 8). The complete genomes were searched through Blastn, revealing that most of the phages were belonged to *Autographiviridae* and *Pootjesviridae* (> 50% coverage and 75% identity). Only 17 phages were assigned to unidentified family due to low coverage and %identity. According to the tree, phage IC12, IG49 and LG08 belong to the same family, *Pootjesviridae*, but are located in different branches with a bootstrap value exceeding 70%, indicating the results are reliable. In addition, the life cycle of these phages was predicted through Bacphlip. A total of 22 phages were classified as temperate phages, showing the presence of integrase, recombinase, or transposase, while the majority of the phages was classified as virulent or lytic (probability of having a virulent lifestyle >0.8). Phages IC12, IG49, and LG08 were classified to have a lytic cycle. A search for virulence factors or antimicrobial resistance genes found that none of the phages contained any of these factors, suggesting that these phages possessed potential lytic ability and are safe for use in agricultural systems (Fernández et al., 2019).

### 3.6 Efficacy of a phage cocktail in reducing *Rhizobium radiobacter* in soil

The lytic ability of the phage cocktail with IC12, IG49, and LG08 to control *R. radiobacter* PL17 in a soil-based substrate was assessed, using an MOI of 100 PFU/CFU as determined by phage lysis efficacy in broth.

**Figure 9:**
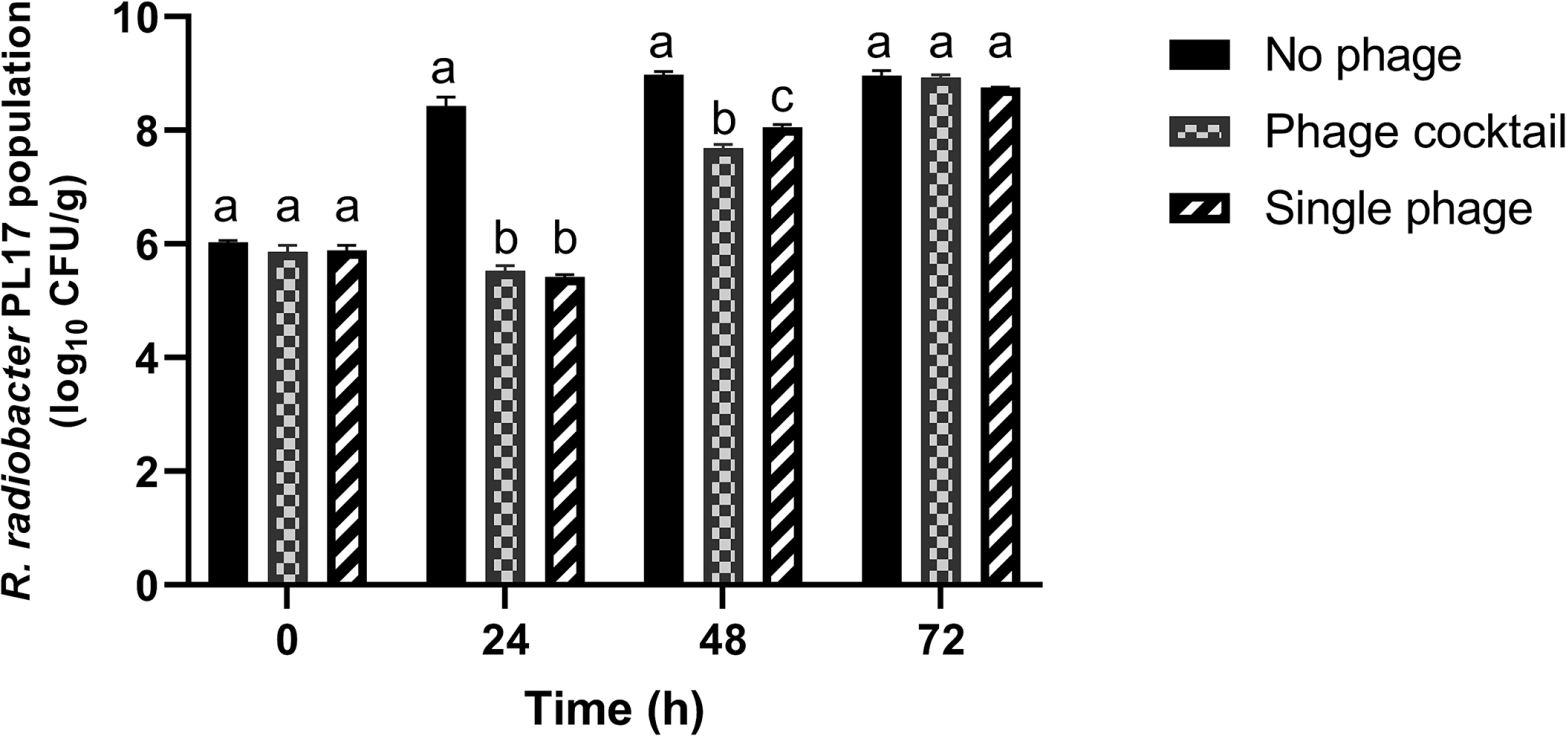
Control of *R. radiobacter* PL17 in a soil-based system with the phage cocktail IC12, IG49 and LG08 and single phage IG49. Data shown are the mean of three replicates ±SD. Lowercase letters indicate a significant difference in the bacterial population number (*p* < 0.05).

Treatment with the phage cocktail resulted in a significant (*p* < 0.05) reduction of 2.9 log_10_ CFU/g from 8.43 log_10_ CFU/g of *R. radiobacter* PL17 after 24 h of treatment (Figure 9). Treatment with a single phage (IG49) also resulted in a significant decrease (*p* < 0.05) in the host cells from 8.43 log_10_ CFU/g to 5.42 log_10_ CFU/g after 24 h of treatment. Furthermore, treatment with the phage cocktail showed no significant difference (*p* > 0.05) when compared to the treatment with a single phage. After 48 h of treatment, the phage cocktail demonstrated a significant reduction (*p* < 0.05) from 8.98 log_10_ CFU/g to 7.69 log_10_ CFU/g of the host cells, while treatment with a single phage showed only a 0.93 log_10_ CFU/g reduction (*p* < 0.05) in the host cells when compared to the no-phage treatment. However, treatment with the phage cocktail or single phage was not significantly different (*p* > 0.05) from the no-phage treatment after 72 h of treatment.

## 4 Discussion

The emergence of stem or crown gall disease in blueberry, especially in highbush varieties, has posed a significant challenge for farmers in British Columbia. Knowledge regarding effectively controlling stem or crown gall disease has been lacking. Current methods involve pruning infected branches and root treatment with *A. radiobacter* K84 (Dygall®) at planting. In addition, use of antibiotics or chemicals like copper to control bacterial diseases is restricted, and their intensive use may lead to the development of copper- and antibiotic-resistant strains through processes like horizontal gene transfer (Song et al., 2021). Moreover, these methods can harm beneficial microbes. Therefore, a phage-based control strategy with high host specificity offers a targeted solution to this issue.

Phages are ubiquitous and can be found in close proximity to their target hosts. In our study, however, *Rhizobium* phages were not found in soil samples collected from blueberry fields infected with stem gall. This can be contributed to several factors, including a long-term storage of soil samples in the laboratory and the negative effect of ice crystals on phages when samples were frozen during storage. In addition, phages can undergo a lysogenic cycle and become temperate phages at low temperatures (Shan et al., 2014). In the fresh soil samples collected around the blueberry plants with stem gall symptoms and processed immediately after collection, no phages were recovered. It could be due to phages potentially binding to soil particles or presented in low concentrations, making them difficult to extract through centrifugation and filtration. Another possibility could be the absence of *R. radiobacter* as a host in the soil, as phages require a host to coexist and propagate; however, *R. radiobacter*, being a soil-inhabitant, is expected to be present in the infected fields. In addition, isolation of phages was unsuccessful from the water samples collected from a drainage ditch in a blueberry farm with stem gall disease. In a study conducted by Boyd et al., *Rhizobium* phages were successfully recovered from sewage (Boyd et al., 1970). Therefore, sewage appears to be a potential source of *Rhizobium* phages, as evidenced by the recovery of 76 phages in this study.

Previous studies have showed that combinations of lytic phages in a cocktail could overcome phage resistance developed by bacteria and significantly enhance the efficacy in controlling pathogens (Ye et al., 2019). In formulating an effective phage cocktail, factors such as lytic ability, high progeny production, and host specificity need to be considered (Merabishvili et al., 2018; Villalpando-Aguilar et al., 2022). In our study, we identified five candidate phages for the formulation of phage cocktail based on the host range assessment and other characteristics. Single-step growth curves were used to determine how fast a phage replicates in the host cell and the number of progenies produced at the time of lysis. Phage IG49 displayed a burst size of 33 phages per infected cell, higher than the other phages tested, while phage LG08 had a low burst size of 4 phages per infected cell. Although an effective phage-host interaction is expected to have a short latent period and a high burst size (Kalatzis et al., 2016), phage LG08 displayed lytic ability across a wide host range, making it a suitable candidate to be used in the phage cocktail treatment.

The long-term stability of the phages is essential for developing a phage-based biocontrol system that can be readily used in agriculture. The optimal temperature for growing highbush blueberries typically ranges from 20 to 25°C, depending on the cultivar (Hao et al., 2019). For blueberries, optimal soil pH falls between 4.2–5.5. A pH beyond this range can affect the growth, physiological metabolism, and yield of blueberries (Zhou et al., 2022). In our study, phages IC12, IG49, AN01, and LG08 demonstrated stability at pH 4, 6, and 8. However, phage LG11 showed a decrease to below the limit of detection (LOD) after 15 days of storage, indicating the instability of the phage over a period of time. Regarding temperature stability, the selected phages remained stable at 4, 22 and 37°C after 30 days of storage. These results suggest that the phages retained their stability within the expected temperature and pH range that is relevant to the growing conditions of highbush blueberry.

Whole-genome sequencing has been recognized as a method to study the genetic elements of phages, providing insights into their characteristics and safety for both human and environmental applications. Based on our study, none of the candidate phages presented an integrase gene, indicating a probability of being a lytic phage greater than 0.9. Lytic phages do not integrate their genetic material into genome of host bacteria, making them suitable for biocontrol applications (Huss and Raman, 2020). Furthermore, the absence of virulent genes and antimicrobial resistance genes in the genomes confirmed their safe use in agricultural. These findings are important to ensure that the phages selected in this study do not contribute to the development of antibiotic resistance. Based on the phylogenetic tree, phage IC12, IG49 and LG08 could be classified as new members of the *Pootjesviridae* family, with more than 93 percent identity to *Agrobacterium* phage OLIVR5 (accession no. NC_055841.1) (Fortuna et al., 2023). According to Fortuna et al. (2023), the morphology of phages in the *Pootjesviridae* family contain a long contractile tail. The TEM study confirmed the morphology of the three phages, IC12, IG49 and LG08, suggesting a structural analogy to the family *Myoviridae*, and the whole-genome sequencing offers a more in-depth understanding regarding taxonomic classification.

From these characteristics and whole-genome sequencing analysis, a combination of two-, three-, and four-phage cocktail was formulated against *R. radiobacter*. *R. radiobacter* strain PL17 was selected as the model strain due to the presence of *virB* gene cascade, identified by Biolog and whole-genome sequencing (unpublished data). This *virB* gene cascade accounts for delivering T-DNA to plant cells and causing gall disease (Li and Christie, 2018). Phage efficacy results indicated that the ideal phage cocktail formulation would be a 3-phage cocktail system, consisting of IG49, LG08, and IC12. Despite the effectiveness of phage LG11 and AN01 against *R. radiobacter* PL17, they were excluded from the final cocktail due to specific reasons. Specifically, phage LG11 was unstable at different pH levels, and phage AN01 had a host range similar to phage LG08. These considerations highlight the importance of carefully selecting phages to ensure the overall effectiveness and environmental stability of the phage cocktail. The decision to opt for a 3-phage cocktail, rather than a higher or lower number of phages, aims to maintain a balance between efficacy and production costs.

The efficacy of a 3-phage cocktail in the control of *R. radiobacter* PL17 was evaluated in pasteurized soil-based system to eliminate unintended variables such as soil microbes or other living organisms. The phage cocktail showed an inhibitory effect against *R. radiobacter*, resulting in approximately a 3-log_10_ CFU/g reduction of *R. radiobacter* PL17 after 24 h and a 1.5 log_10_ CFU/g reduction after 48 h. These results showed that the phages successfully interacted with the bacterial host in the soil-based system. While previous reports suggested that cocktail of phages can synergistically enhance the lytic ability against host cells (Glonti and Pirnay, 2022), our study tested a single phage IG49 against *R. radiobacter* PL17 in the soil-based system. Interestingly, the log reduction of bacterial host cells by the single phage was not significantly different when compared to the efficacy of the 3-phage cocktail. A previous study showed that a single phage (OLIVR1) had the ability to reduce *Agrobacterium* host to below the limit of detection in hydroponic solution, suggesting the potential of phage application in hydroponic greenhouse systems (Fortuna et al., 2023). However, compared to our soil-based system, the effectiveness of phage application could be reduced due to factors such as a high concentration of the bacterial host, designed to represent a highly infected blueberry field, or the composition of the growth substrates. The hydroponic nutrient solution used in the study conducted by Fortuna et al. provided more nutrients when compared to 0.1% peptone and soil solution used in this study. In addition, phage-host interaction plays an important role in phage treatment. In our system, soil characteristics may limit the mobility of bacteria due to water absorption within soil particles. Although *R. radiobacter* is motile, most of phages are non-motile, which limit the interaction between phage and host (Li et al., 2020).

## 5 Conclusion

The cocktail of the three-phage system comprising of IC12, IG49 and LG08 was chosen based on their efficacy, stability, and the presence or absence of essential genes. This selected phage system exhibited significant inhibitory ability against *R. radiobacter* in a soil/peat-based system, showing a great potential for preventing stem gall disease of blueberry. Further studies on the assessment of efficacy of the phage system against *R. radiobacter* in stem/crown gall *in planta* and infected blueberry fields are crucial to confirm the effectiveness of this biocontrol agent. Overall, the approach discussed is effective for conceptualizing formulation and commercialization of the bacteriophage system, offering potential benefits to the blueberry industry.

## Data availability statement

The sequences of phage IC12, IG49 and LG08 have been deposited in the GenBank under accession numbers of PP417939, PP429226, and PP429227, respectively.

## Author contributions

BC: Formal analysis, Investigation, Methodology, Visualization, Writing – original draft. SS: Conceptualization, Funding acquisition, Resources, Writing – review & editing. SW: Conceptualization, Funding acquisition, Project administration, Resources, Supervision, Writing – review & editing.
