# Supplementary material for: Isolation and characterization of bacteriophages for controlling Rhizobium radiobacter – causing stem and crown gall of highbush blueberry
Source: Front Microbiol. 2024 Aug 2;15:1437536. doi: 10.3389/fmicb.2024.1437536 (PMC11328917; doi:10.3389/fmicb.2024.1437536)
Supplement: Supplementary file 1 [file Data_Sheet_1.pdf]

Supplementary Material

Isolation and Characterization of Bacteriophages for Controlling *Rhizobium radiobacter* - Causing Stem and Crown Gall of Highbush Blueberry

Bowornnan Chantapakul[1*], Siva Sabaratnam[2], Siyun Wang[1]

*** Correspondence:** Bowornnan Chantapakul: bchantap@mail.ubc.ca

1 Supplementary Figures

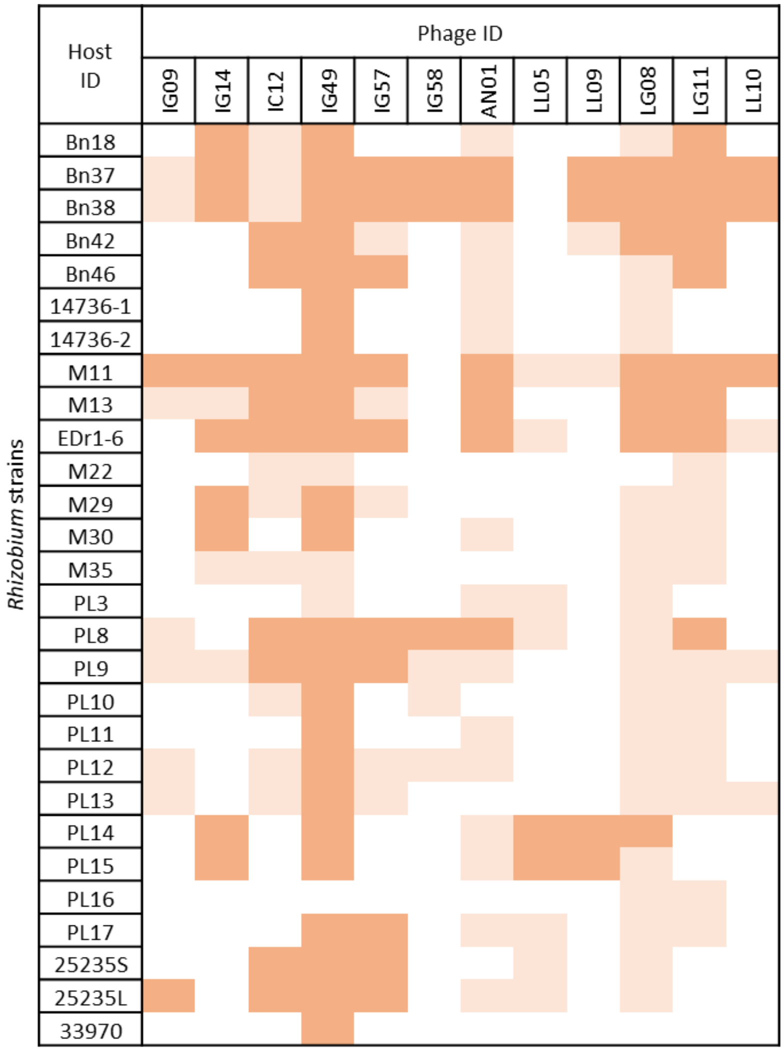

Supplementary Figure 1. Heatmap of a selected individual phage for efficacy against each *R. radiobacter* on the broth system after 72 h. at 25°C (☐ = no inhibition, ▪ = delay the growth, ▪ = complete inhibition).

A

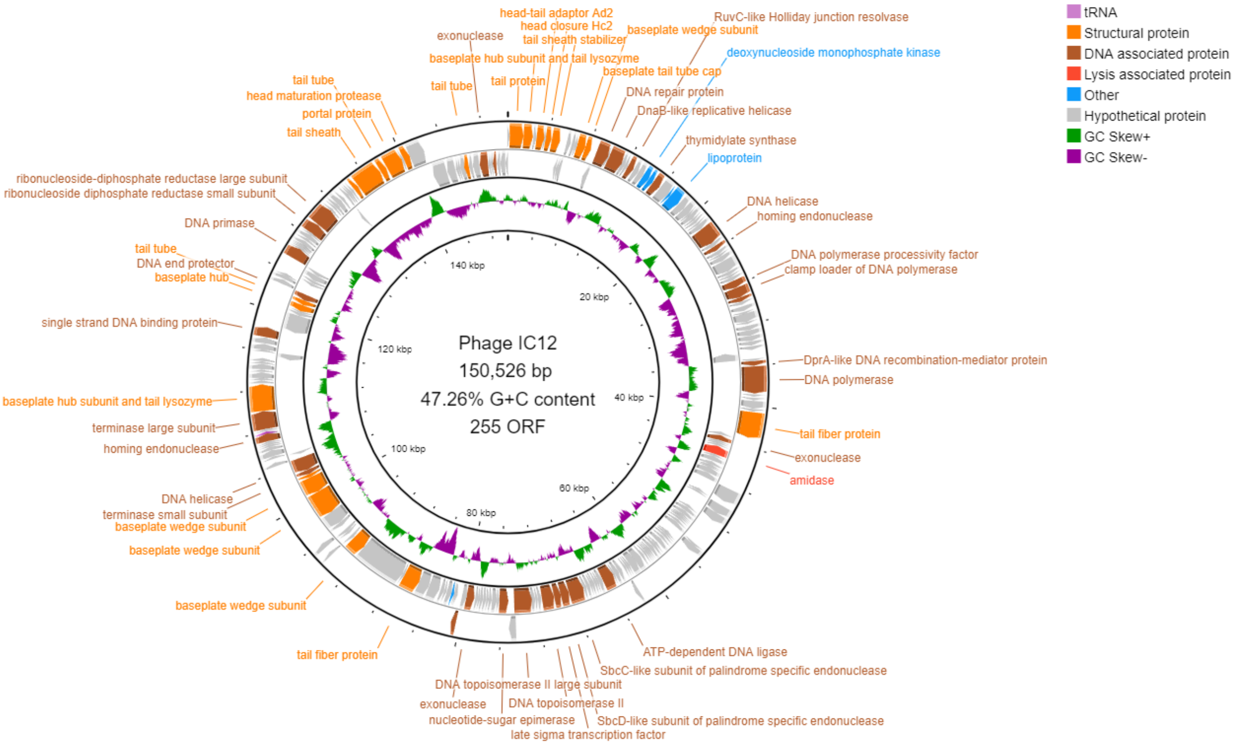
tRNA
Structural protein
DNA associated protein
Lysis associated protein
Other
Hypothetical protein
GC Skew+
GC Skew-
Phage IC12
150,526 bp
47.26% G+C content
255 ORF
20 kbp
40 kbp
60 kbp
80 kbp
100 kbp
120 kbp
140 kbp
head-tail adaptor Ad2
head closure Hc2
tail sheath stabilizer
exonuclease
baseplate hub subunit and tail lysozyme
RuvC-like Holliday junction resolvase
baseplate wedge subunit
deoxynucleoside monophosphate kinase
baseplate tail tube cap
tail tube
tail protein
DNA repair protein
DnaB-like replicative helicase
thymidylate synthase
lipoprotein
tail tube
head maturation protease
portal protein
tail sheath
ribonucleoside-diphosphate reductase large subunit
ribonucleoside diphosphate reductase small subunit
DNA primase
tail tube
DNA end protector
baseplate hub
single strand DNA binding protein
baseplate hub subunit and tail lysozyme
terminase large subunit
homing endonuclease
DNA helicase
terminase small subunit
baseplate wedge subunit
baseplate wedge subunit
baseplate wedge subunit
tail fiber protein
DNA helicase
homing endonuclease
DNA polymerase processivity factor
clamp loader of DNA polymerase
DprA-like DNA recombination-mediator protein
DNA polymerase
tail fiber protein
exonuclease
amidase
ATP-dependent DNA ligase
SbcC-like subunit of palindrome specific endonuclease
DNA topoisomerase II large subunit
exonuclease
DNA topoisomerase II
nucleotide-sugar epimerase
SbcD-like subunit of palindrome specific endonuclease
late sigma transcription factor

B

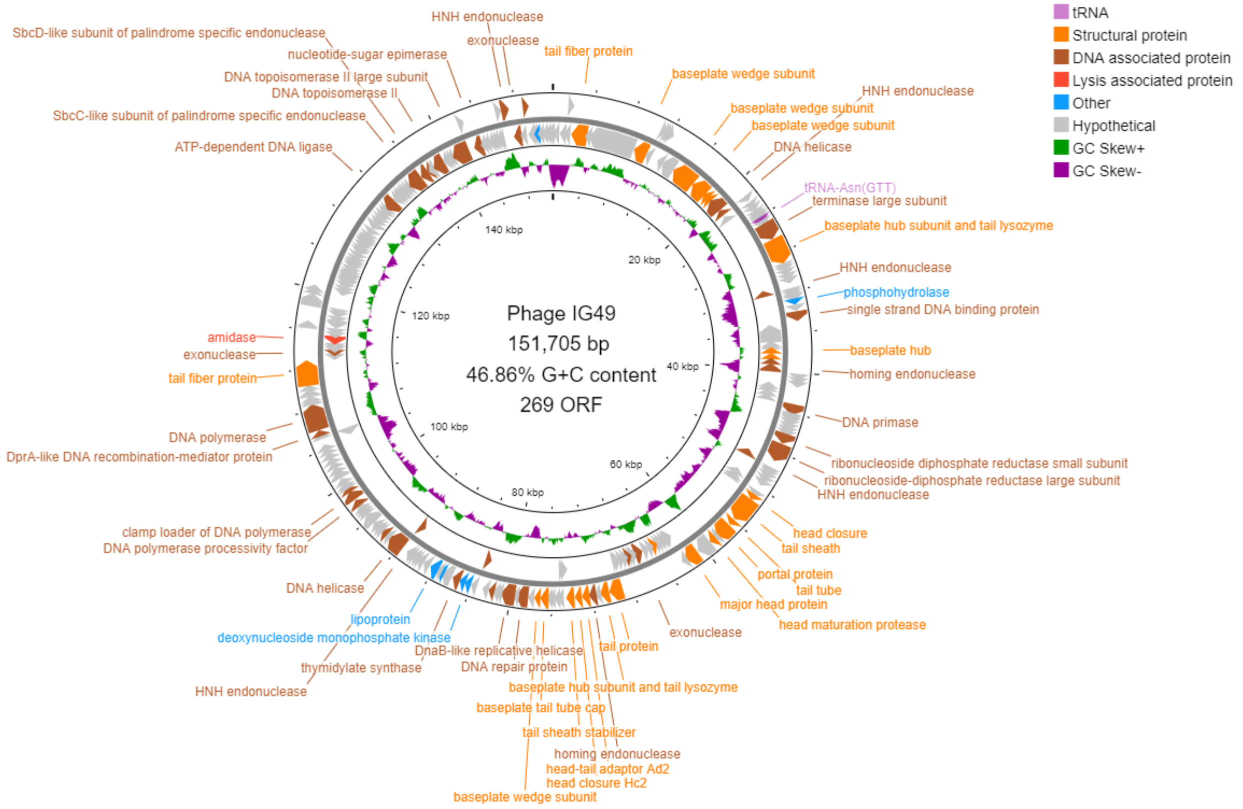
tRNA
Structural protein
DNA associated protein
Lysis associated protein
Other
Hypothetical
GC Skew+
GC Skew-
Phage IG49
151,705 bp
46.86% G+C content
269 ORF
20 kbp
40 kbp
60 kbp
80 kbp
100 kbp
120 kbp
140 kbp
HNH endonuclease
SbcD-like subunit of palindrome specific endonuclease
exonuclease
nucleotide-sugar epimerase
tail fiber protein
DNA topoisomerase II large subunit
DNA topoisomerase II
baseplate wedge subunit
SbcC-like subunit of palindrome specific endonuclease
HNH endonuclease
baseplate wedge subunit
baseplate wedge subunit
ATP-dependent DNA ligase
DNA helicase
tRNA-Asn(GTT)
terminase large subunit
baseplate hub subunit and tail lysozyme
HNH endonuclease
phosphohydrolase
single strand DNA binding protein
amidase
exonuclease
baseplate hub
tail fiber protein
homing endonuclease
DNA polymerase
DNA primase
DprA-like DNA recombination-mediator protein
ribonucleoside diphosphate reductase small subunit
ribonucleoside-diphosphate reductase large subunit
HNH endonuclease
clamp loader of DNA polymerase
DNA polymerase processivity factor
head closure
tail sheath
portal protein
tail tube
DNA helicase
major head protein
head maturation protease
lipoprotein
deoxynucleoside monophosphate kinase
exonuclease
DnaB-like replicative helicase
tail protein
thymidylate synthase
DNA repair protein
HNH endonuclease
baseplate hub subunit and tail lysozyme
baseplate tail tube cap
tail sheath stabilizer
homing endonuclease
head-tail adaptor Ad2
head closure Hc2
baseplate wedge subunit

C

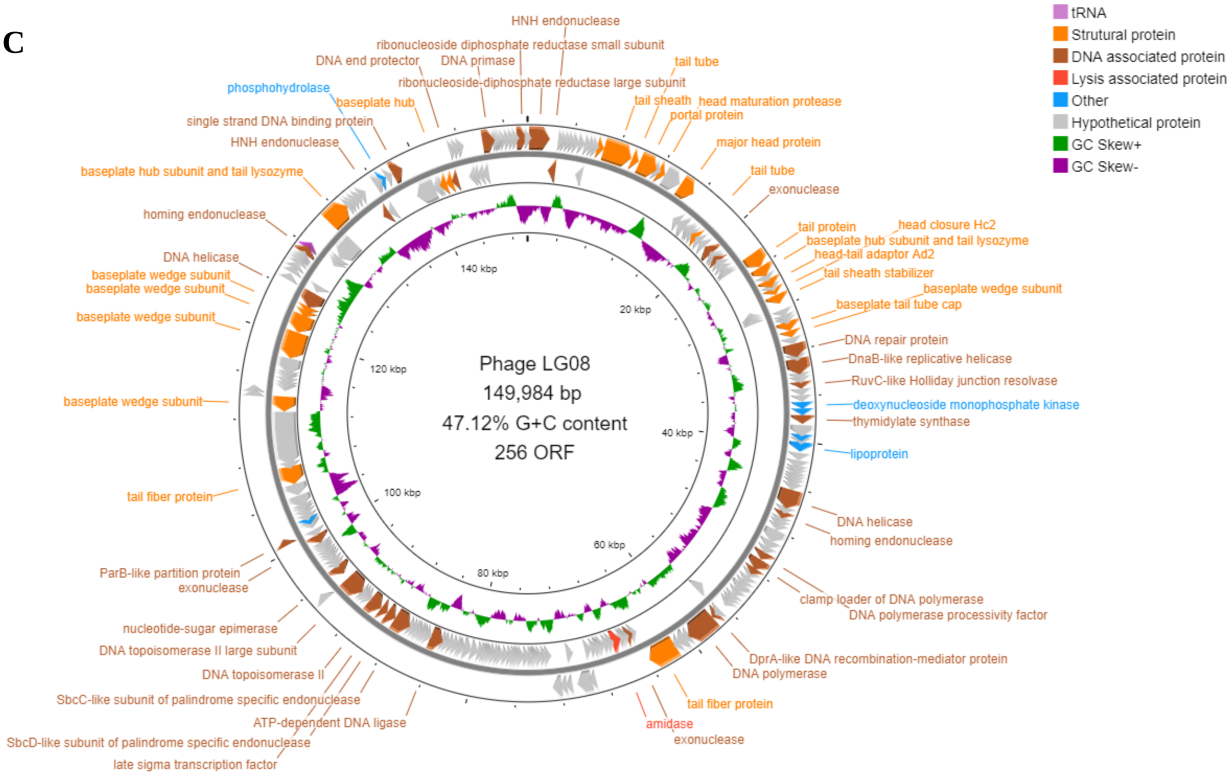

Supplementary Figure 2. Genome map of phages **(A)** IC12, **(B)** IG49 and **(C)** LG08. The inner ring displays GC skew plot and the outer ring displays the ORFs transcribed in clockwise or counterclockwise direction represented by different colored arrows based on their gene functions.
